# The reliability of and agreement between devices used to measure eccentric hamstring strength: a systematic review protocol

**DOI:** 10.1186/s13643-022-02070-8

**Published:** 2022-09-23

**Authors:** Daniel Torpey, Eoghan Murray, Tom Hughes, Jamie Sergeant, Michael Callaghan

**Affiliations:** 1 Manchester United Football Club, AON Training Complex, Birch Rd off Isherwood, Road, Manchester, M31 4BH UK; 2 Academy Medical Department, Manchester United Football Club, Manchester, UK; 3 Department of Health Professions, Manchester Metropolitan University, Manchester, UK; 4 Centre for Biostatistics, Manchester Academic Health Science Centre, University of Manchester, Manchester, UK; 5 Department of Health Professions, Manchester Metropolitan University, Manchester, UK

**Keywords:** Isokinetic, Dynamometry, Measurement, Nordic, Test-retest, Repeated measures

## Abstract

**Background:**

Isokinetic dynamometry (IKD) is considered as the gold standard method of eccentric hamstring strength measurement, but other devices are more portable, cost-effective, provide real-time data and are thus better suited to the mass testing required in sport.

This review aims to synthesise the evidence related to the reliability of and agreement between devices that measure eccentric hamstring strength and isokinetic dynamometers in adults.

**Methods:**

The MEDLINE, EMBASE, PubMed, CINAHL and Sport Discus databases, alongside a search of grey and pre-print literature (from inception to 2021), are used. Forward and backward snowballing will also be used. Studies will be included if the reliability and/or agreement between devices used to quantify eccentric hamstring strength in healthy, recreationally active or amateur/elite sportspeople has been investigated. Studies will be excluded if (1) participants were injured or unwell at the time of testing and (2) concentric strength measurements or if non-hamstring muscle groups were investigated.

The COnsenus-based Standards for the selection of health Measurement INstruments (COSMIN) tool will be used to assess the quality of reporting of included studies.

If possible, data will be pooled and a meta-analysis and/or meta-regression may be performed if appropriate. We will aim to conduct a narrative synthesis using an adapted Grading of Recommendation, Assessment, Development and Evaluation (GRADE).

**Discussion:**

This systematic review will aim to analyse the reliability of devices that measure eccentric hamstring strength, and the agreement of these devices with isokinetic dynamometers when used in an adult population. It is anticipated that the results of this review could be used to inform clinicians regarding suitable devices that can be employed to monitor eccentric hamstring strength in clinical practice.

No ethics approval is required. It is anticipated that this review will be submitted to a leading peer-reviewed journal in this field for publication consideration.

**Systematic review registration:**

www.researchregistry.com (reviewregistry1070)

**Supplementary Information:**

The online version contains supplementary material available at 10.1186/s13643-022-02070-8.

## Introduction

### Rationale

Hamstring strain injury (HSI) is one of the most common injuries in sports [1]. HSIs account for 12–15% of all injuries that occur in football (soccer), Australian Rules football and American Football [2–4]. This can result in a significant loss of training and competition time and can affect the quality of life of injured athletes [1]. Additionally, HSIs have a high risk of recurrence, which can affect up to 22% of cases in soccer [5] and 34% in Australian Rules football [6].

HSIs typically occur in sports where running and skilled movements at high speed, kicking or combined hip flexion and knee flexion movements are required [7]. In particular, the hamstrings are more susceptible to injury in the terminal stance and terminal swing phases of running [8, 9]. This is because the hamstrings work eccentrically to decelerate knee extension during these phases, and when combined with a hip flexion position, this can produce a significant elongation stress to the hamstring musculature [10, 11]. Consequently, eccentric strengthening has formed a key role in athlete conditioning and injury mitigation strategies. Indeed, eccentric strengthening has been shown to reduce the risk of hamstring injuries in cohorts of soccer players at amateur and elite levels [12–14].

To quantify changes in eccentric strength that result from such conditioning strategies, it is suggested that testing procedures are implemented as part of ongoing hamstring strength monitoring rather than just at baseline screening [15]. It has also been shown in a cohort of athletes with hamstring injuries that testing hamstring muscle strength at regular intervals can be meaningful to inform the progression of loading during rehabilitation and return to participation in sport [16].

Isokinetic dynamometry (IKD) devices are often cited as the gold standard device for all forms of muscle strength testing [17, 18]. IKDs demonstrate thorough standardization, can perform isokinetic and isometric testing, and can test muscle groups through a large range of motion, at different velocities. IKDs achieve this without having to account for a strength imbalance between the participant and the assessor [19]. However, their application is limited due to purchase and maintenance costs, the considerable time required to complete assessments and a lack of portability [20]. Alternatively, other devices are available that are portable, cost-effective, provide real-time data and thus better suited to mass testing, which is typically required in sports. These include hand-held dynamometers (HHDs) and devices used to measure eccentric strength during the Nordic exercise and have previously been deemed reliable [21, 22].

Previous systematic reviews in this area [23, 24] have assessed HHD reliability compared to IKD, but the studies only involved isometric and concentric muscle strength testing procedures. Furthermore, Claudino et al. [25] reviewed all devices that test eccentric hamstring strength, but the focus of the review did not include the reliability or agreement between these devices. Currently, there is a gap in the literature for the amalgamation and evaluation of reliability and agreement data in this area. Providing this information may identify the most reliable devices used to test eccentric hamstring strength. This could assist with clinical reasoning areas such as injury rehabilitation progression, return to play decisions [16] and in-season hamstring fatigue monitoring [26].

### Objectives

This review therefore aims to (a) present the reliability (interrater and intrarater) of all devices that measure eccentric hamstring strength and/or (b) present the agreement these devices have with IKDs.

## Methods

### Eligibility criteria

The following criteria will be grouped together in the PICO (population, intervention, comparator, outcome) format. This strategy will help to identify information that is relevant to the research question [27]. For this review, the comparator group will not be used, to ensure that studies are not excluded if they do not include a direct comparison with IKDs. However, it is anticipated that the number of studies with a direct comparison to IKD is low. Hence, the dual purpose of reviewing all studies that analyse the reliability of devices and/or provide the level of agreement with IKDs.

### Population

Studies investigating adults (≥16 years.) and cohorts considered recreationally active, athletic, uninjured or healthy. Study cohorts with known musculoskeletal injuries or neurological/medical conditions will not be considered.

### Intervention

Devices that measure eccentric hamstring strength will be included e.g. hand-held dynamometers, isokinetic dynamometers (including but not limited to Cybex, KinCom, Biodex, Primus or similar devices) and devices that test eccentric hamstring strength during the Nordic exercise. The device output measurement from such devices is peak/average force (e.g. Kg, N) or peak/average torque (e.g. Nm). The devices listed may each test hamstring strength at varying velocities, but the key element of the review is the eccentric action of the hamstrings. It is therefore essential to include all devices, regardless of testing velocity, provided there is an eccentric element.

Studies will be excluded if they utilise devices that only measure concentric or isometric hamstring strength (i.e. portable fixed dynamometers) or test other muscle groups.

### Outcome

Studies will be eligible if during the reliability analysis they consider any of the following outcomes: (1) intraclass correlation coefficients (ICC), which quantifies the reliability of measurements or ratings; (2) standard error of measurements (SEM), which quantifies absolute consistency, provides the precision of a score and allows the construct of the confidence interval (CI) for scores; and (3) minimal detectable changes (MDC), which are statistical estimates of the smallest amount of change that can be detected by a measure that corresponds to a noticeable change in ability [28]. In addition, during the analysis of agreement between devices, they include 95% limits of agreement (LoA) using the Bland and Altman method [29]. It is anticipated that studies may include reliability and/or agreement analysis.

Studies should include a period of 2 weeks or less between interval measurements. This interval is sufficient so that a learning effect does not occur, but not so much that the construct being tested (i.e. muscle strength) could change [30].

#### Patient and public involvement

Patients and/or the public are not involved in this review.

### Information sources

The following databases will be searched for relevant published studies: (1) MEDLINE, (2) EMBASE, (3) PubMed, (4) CINAHL and (5) Sport Discus from inception to 2021. This will be supplemented by a search of grey literature and unpublished research via search engines (Google Scholar), forward and backward snowballing and pre-print search via medrxiv.org. All searches will be limited to full-text articles that have investigated human participants, in the English language only. Conference abstracts will be excluded.

### Search strategy

This search has been finalised, and it will be adapted for use in the other databases (Table 1). In the final stages of the review, the search will be repeated to ensure that all relevant studies have been captured. See Supplementary file 1 for additional database search strategies.

**Table 1:** The MEDLINE database search strategy

	MEDLINE search strategy – 11th Aug 2022
Population	1.Young Adult/ or Adult/ 2. athlet*.mp. 3. 1 or 2
Intervention	4. nordic*.mp. 5. (Nordic adj3 exercise).mp. [mp=title, abstract, original title, name of substance word, subject heading word, floating sub-heading word, keyword heading word, organism supplementary concept word, protocol supplementary concept word, rare disease supplementary concept word, unique identifier, synonyms] 6. (Nordic adj3 device).mp. [mp=title, abstract, original title, name of substance word, subject heading word, floating sub-heading word, keyword heading word, organism supplementary concept word, protocol supplementary concept word, rare disease supplementary concept word, unique identifier, synonyms] 7. (Strength adj2 device).mp. [mp=title, abstract, original title, name of substance word, subject heading word, floating sub-heading word, keyword heading word, organism supplementary concept word, protocol supplementary concept word, rare disease supplementary concept word, unique identifier, synonyms] 8. (Eccentric adj3 device).mp. [mp=title, abstract, original title, name of substance word, subject heading word, floating sub-heading word, keyword heading word, organism supplementary concept word, protocol supplementary concept word, rare disease supplementary concept word, unique identifier, synonyms] 9. (Eccentric adj3 strength).mp. [mp=title, abstract, original title, name of substance word, subject heading word, floating sub-heading word, keyword heading word, organism supplementary concept word, protocol supplementary concept word, rare disease supplementary concept word, unique identifier, synonyms] 10. "Measurement device".mp. 11. Nordbord.mp. 12. "handheld dynamomet*".mp. 13. "digital dynamomet*".mp. [mp=title, abstract, original title, name of substance word, subject heading word, floating sub-heading word, keyword heading word, organism supplementary concept word, protocol supplementary concept word, rare disease supplementary concept word, unique identifier, synonyms] 14. Lafayette.mp. 15. Myometer.mp. 16. ActivForce.mp. [mp=title, abstract, original title, name of substance word, subject heading word, floating sub-heading word, keyword heading word, organism supplementary concept word, protocol supplementary concept word, rare disease supplementary concept word, unique identifier, synonyms]. 17. MicroFET2.mp. [mp=title, abstract, original title, name of substance word, subject heading word, floating sub-heading word, keyword heading word, organism supplementary concept word, protocol supplementary concept word, rare disease supplementary concept word, unique identifier, synonyms]. 18. Biodex.mp. 19. Primus.mp. 20. Cybex.mp. 21. KinCom.mp. 22. Isokinetic device.mp. 23. 4 or 5 or 6 or 7 or 8 or 9 or 10 or 11 or 12 or 13 or 14 or 15 or 16 or 17 or 18 or 19 or 20 or 21 or 22.
Outcome	24. Reliability.mp. 25. Agreement.mp. 26. Test-retest.mp 27. Repeated-measures.mp. 28. 24 or 25 or 26 or 27
	29. 3 and 23 and 27 30. limit 28 to (English language and humans)

### Study records

#### Data management

The main author (DT) will import the literature search results to Mendeley (Elsevier, Version 1.19.5, London) and utilise the de-duplicator tool to remove duplicates. A Preferred Reporting Items for Systematic reviews and Meta-Analyses (PRISMA) flowchart will be developed to demonstrate the process of the search and filtering of studies [31]. See Supplementary file 2.

#### Data selection and collection process

Two reviewers (DT and EM) will independently screen the titles and abstracts generated from the search against the eligibility criteria. If it is uncertain as to whether a study meets the eligibility criteria, the full text of each will be obtained, which will be used to screen against the eligibility criteria. If consensus cannot be reached between reviewers, an independent adjudicator (MC) will be used. The reasons for exclusion will be recorded and presented in the final manuscript. None of the review authors will be blinded to the journal and study titles, authors or objectives of any studies that are under consideration.

A standardised form derived from the COnsensus-based Standards for the Selection of health Measurement INstruments (COSMIN) tool for studies on reliability or measurement error will be utilised by the main author (DT) to extract data from each eligible study [32–34]. This data will be verified by a second author (EM), and if an agreement cannot be reached, then an independent adjudicator (MC) will be involved to provide a resolution. If required, the study authors will be contacted for further information.

#### Data items

The data items extracted from selected articles will include (1) the name of the outcome measurement instrument; (2) the version of the outcome measurement instrument or way of operationalisation of the measurement protocol; (3) the construct measured by the measurement instrument; (4) the estimates of reliability, measurement error, agreement and the associated confidence intervals; (5) the components of the measurement instrument that were repeated; (6) the source of variation that will be varied; and (7) the patient population. In cases of missing information, we will attempt to contact the study authors directly.

#### Outcomes and prioritization

This review will consider the following outcomes: (1) intraclass correlation coefficients (ICC), which quantifies the reliability of measurements or ratings; (2) standard error of measurements (SEM), which quantifies absolute consistency, provides the precision of a score and allows the construct of the confidence interval (CI) for scores; and (3) minimal detectable changes (MDC), which are statistical estimates of the smallest amount of change that can be detected by a measure that corresponds to a noticeable change in ability [28]; and (4) 95% limits of agreement (LoA) between devices using the Bland and Altman method [29].

#### Risk of bias

The COSMIN tool will be used to assess the quality of reporting of included studies, across nine reliability criteria and eight measurement error criteria. These criteria are graded ‘very good’, ‘adequate’, ‘doubtful’, ‘inadequate’ or ‘N/A’. The quality of each study will be rated with a worst-score-count method to determine the risk of bias [32–34].

The main author (DT) and one other author (EM) will independently appraise each study and then discussed together afterwards. Any disagreements will be resolved by an independent adjudicator (MC). Only those studies achieving ‘very good’ or ‘adequate’ on the overall rating scale will be included in the final review.

#### Data synthesis

Studies investigating similar measurement devices and outcomes will be grouped and evaluated for heterogeneity, across the domains of (1) risk of bias, (2) population and (3) statistical analysis. Data will be presented using text and tables to summarise the characteristics and findings, as well as exploring the relationship within and between the included studies [35].

The ICC is a relative measure of reliability and is reflective of the ability of a test to differentiate between different individuals. However, the ICC is context-specific which is highlighted by the fact the magnitude of the ICC depends on the between-subject variability. Conversely, the SEM is not affected by between subjects’ variability [28]. It is an index of absolute consistency and quantifies the precision of individual scores on a test [36]. If there is subject homogeneity, it is difficult to differentiate between subjects using the ICC, even if the measurement error is small. Therefore, an examination of the SEM along with the ICC is required [28]. In this review, it is anticipated that the studies analysed will use a combination of reliability measures.

A meta-analysis will be considered if there are two or more studies that have a low risk of bias, the same reliability statistic, the same type of device and the same population. The process will be conducted using Stata 16.1 (StataCorp LLC, Texas, USA). Heterogeneity of studies will be assessed by using the I2 index and if it is high (I2>50% or considerable differences observed in study characteristics exist), a meta-analysis will not be performed and a narrative synthesis will be conducted. The factors causing heterogeneity may also be evaluated using subgroup analysis or sensitivity analysis if possible (see Table 2 for pre-defined subgroups). If there is a sufficient sample size of studies, we plan to perform a random-effects meta-analysis following the DerSimonian and Laird approach [37].

**Table 2:** The subgroups that will be analysed in the order of priority

Subgroup	Category
Cohort age range	e.g. 16 to 40 years/40+ years
Sport played by cohort	e.g. Soccer/Australian Rules Football/Rugby/Athletics/Hockey, etc.
Biological sex	e.g. male/female
Cohort setting	e.g. professional or amateur athletes/sporting population or general population

Limits of agreement data will be analysed via a narrative synthesis and presented using both text and tables. A meta-regression will be considered if there are more than ten studies in the meta-analysis. The subgroups shown in Table 2 will be used, as an extension to subgroup analysis, to investigate the effects of the continuous and categorical characteristics on the study outcomes.

#### Confidence in cumulative evidence

The strength of evidence found during the review will be evaluated using a modified Grading of Recommendation, Assessment, Development and Evaluation (GRADE) framework, and a summary of findings table will be created. The GRADE approach is a system for evaluating the quality of evidence for outcomes reported in systematic reviews. Evidence is classified into four levels of quality: high, moderate, low and very low [38]. The GRADE framework was not designed specifically for use with reliability studies, so an adapted version will be used (Supplementary file 3).

#### Additional analyses

Sensitivity analyses will be performed to explore the source of heterogeneity by using quality components such as published (i.e. peer-reviewed) vs unpublished (i.e. pre-prints) data [39].

#### Meta-bias(es)

We intend to use appropriate graphical methods and statistical methods to assess for small study effects, such as, funnel plots to assess for publication bias and the COSMIN tool to assess selective outcome reporting. We plan to account for publication bias by performing a search of grey literature and unpublished research via search engines (Google Scholar and MedRxiv.org).

## Discussion

This systematic review will aim to analyse the reliability of devices that measure eccentric hamstring strength and the agreement of these devices with isokinetic dynamometers when used in an adult population. It is anticipated that the results of this review could be used to inform clinicians regarding suitable devices that can be employed to test eccentric hamstring strength in practice.

## Supplementary Information

**Additional file 1.** Database Search strategies.

**Additional file 2.** PRISMA flowpart.

**Additional file 3.** Guide for judgement of modified GRADE synthesis.

## Data Availability

The datasets used and/or analysed during the current study are available from the corresponding author upon reasonable request.
