# Supplementary material for: The reliability of and agreement between devices used to measure eccentric hamstring strength: a systematic review protocol
Source: Syst Rev. 2022 Sep 23;11:204. doi: 10.1186/s13643-022-02070-8 (PMC9502956; doi:10.1186/s13643-022-02070-8)
Supplement: Supplementary file 1 — Additional file 1. Database Search strategies. [file 13643_2022_2070_MOESM1_ESM.docx]

Database Search strategies

Medline (OVID) – 1946 to July 2022

S1. (young adult or adult).mp. [mp=title, abstract, original title, name of substance word, subject heading word, floating sub-heading word, keyword heading word, organism supplementary concept word, protocol supplementary concept word, rare disease supplementary concept word, unique identifier, synonyms]

S2. athlet*.mp.

S3. 1 or 2

S4. nordic*.mp.

S5. (Nordic adj3 exercise).mp. [mp=title, abstract, original title, name of substance word, subject heading word, floating sub-heading word, keyword heading word, organism supplementary concept word, protocol supplementary concept word, rare disease supplementary concept word, unique identifier, synonyms]

S6. (Nordic adj3 device).mp. [mp=title, abstract, original title, name of substance word, subject heading word, floating sub-heading word, keyword heading word, organism supplementary concept word, protocol supplementary concept word, rare disease supplementary concept word, unique identifier, synonyms]

S7. (Strength adj2 device).mp. [mp=title, abstract, original title, name of substance word, subject heading word, floating sub-heading word, keyword heading word, organism supplementary concept word, protocol supplementary concept word, rare disease supplementary concept word, unique identifier, synonyms]

S8. (Eccentric adj3 device).mp. [mp=title, abstract, original title, name of substance word, subject heading word, floating sub-heading word, keyword heading word, organism supplementary concept word, protocol supplementary concept word, rare disease supplementary concept word, unique identifier, synonyms]

S9. (Eccentric adj3 strength).mp. [mp=title, abstract, original title, name of substance word, subject heading word, floating sub-heading word, keyword heading word, organism supplementary concept word, protocol supplementary concept word, rare disease supplementary concept word, unique identifier, synonyms]

S10. "Measurement device".mp.

S11. Nordbord.mp.

S12. "Handheld dynamometer".mp.

S13. "Digital dynamometer".mp.

S14. Lafayette.mp.

S15. Myometer.mp.

S16. ActivForce.mp. [mp=title, abstract, original title, name of substance word, subject heading word, floating sub-heading word, keyword heading word, organism supplementary concept word, protocol supplementary concept word, rare disease supplementary concept word, unique identifier, synonyms]

S17. MicroFET2.mp. [mp=title, abstract, original title, name of substance word, subject heading word, floating sub-heading word, keyword heading word, organism supplementary concept word, protocol supplementary concept word, rare disease supplementary concept word, unique identifier, synonyms]

S18. Biodex.mp.

S19. Primus.mp.

S20. Cybex.mp.

S21. KinCom.mp.

S22. Isokinetic device.mp.

S23. 5 or 6 or 7 or 8 or 9 or 10 or 11 or 12 or 13 or 14 or 15 or 16 or 17 or 18 or 19 or 20 or 21 or 22

S24. Reliability.mp.

S25. Agreement.mp.

S26. Test-retest.mp.

S27. Repeated-measures.mp.

S28. 24 or 25 or 26 or 27

S29. 3 and 23 and 28

S30. limit 29 to (english language and humans)

_______________________________

CINAHL Plus (EBSCOhost) 1937-July 2022

S1. (MH "Young Adult/EV") OR "young adult" OR (MH "Adult/EV")

S2. (MH "Athletes, Professional/EV") OR (MH "Athletes, College/EV") OR (MH "Athletes,

Amateur/EV") OR (MH "Athletes, Female/EV") OR (MH "Athletes, Male/EV") OR (MH

"Athletes, High School/EV") OR (MH "Athletes, Elite/EV") OR "athlet*"

S3. S1 OR S2

S4. "nordic*"

S5 "nordic*exercise"

S6 "nordic*device"

S7 "strength*device"

S8 "eccentric*device"

S9 "eccentric*strength"

S10 "measurement device"

S11 "Nordbord"

S12 "handheld dynamomet*"

S13 "digital dynamomet*"

S14 "Lafayette"

S15 "Myometer"

S16 "ActivForce"

S17 "MicroFET2"

S18 "Biodex"

S19 "Primus"

S20 "Cybex"

S21 "KinCom"

S22 "Isokinetic device"

S23 S4 OR S5 OR S6 OR S7 OR S8 OR S9 OR S10 OR S11 OR S12 OR S13 OR S14 OR S15 OR S16 OR

S17 OR S18 OR S19 OR S20 OR S21 OR S22)

S24 (MH "Reliability") OR "reliability" OR (MH "Interrater Reliability") OR (MH "Intrarater

Reliability") OR (MH "Equipment Reliability")

S25 "agreement"

S26 (MH "Test-Retest Reliability") OR "test-retest"

S27 (MH "Repeated Measures") OR "repeated-measures"

S28 (S24 OR S25 OR S26 OR S27)

S29 (S3 AND S23 AND S28)

S30 (S3 AND S23 AND S28) Limiters - English Language; Human

______________________________________

Embase (OVID online) 1974 – July 2022

S1. (young adult or adult).mp. [mp=title, abstract, heading word, drug trade name, original title,

device manufacturer, drug manufacturer, device trade name, keyword heading word,

floating subheading word, candidate term word]

S2. athlet*.mp.

S3. 1 or 2

S4. nordic*.mp.

S5. (Nordic adj3 exercise).mp. [mp=title, abstract, heading word, drug trade name, original title,

device manufacturer, drug manufacturer, device trade name, keyword heading word,

floating subheading word, candidate term word]

S6. (Nordic adj3 device).mp. [mp=title, abstract, heading word, drug trade name, original title,

device manufacturer, drug manufacturer, device trade name, keyword heading word,

floating subheading word, candidate term word]

S7. (Strength adj2 device).mp. [mp=title, abstract, heading word, drug trade name, original title,

device manufacturer, drug manufacturer, device trade name, keyword heading word,

floating subheading word, candidate term word]

S8. (Eccentric adj3 device).mp. [mp=title, abstract, heading word, drug trade name, original title,

device manufacturer, drug manufacturer, device trade name, keyword heading word,

floating subheading word, candidate term word]

S9. (Eccentric adj3 strength).mp. [mp=title, abstract, heading word, drug trade name, original

title, device manufacturer, drug manufacturer, device trade name, keyword heading word,

floating subheading word, candidate term word]

S10. "Measurement device".mp.

S11. Nordbord.mp.

S12. "Handheld dynamometer".mp.

S13. "Digital dynamometer".mp.

S14. Lafayette.mp.

S15. Myometer.mp.

S16. ActivForce.mp. [mp=title, abstract, heading word, drug trade name, original title, device

manufacturer, drug manufacturer, device trade name, keyword heading word, floating

subheading word, candidate term word]

S17. MicroFET2.mp. [mp=title, abstract, heading word, drug trade name, original title, device

manufacturer, drug manufacturer, device trade name, keyword heading word, floating

subheading word, candidate term word]

S18. Biodex.mp.

S19. Primus.mp.

S20. Cybex.mp.

S21. KinCom.mp.

S22. Isokinetic device.mp.

S23. 5 or 6 or 7 or 8 or 9 or 10 or 11 or 12 or 13 or 14 or 15 or 16 or 17 or 18 or 19 or 20 or 21 or

22

S24. Reliability.mp.

S25. Agreement.mp.

S26. Test-retest.mp.

S27. Repeated-measures.mp.

S28. 24 or 25 or 26 or 27

S29. 3 and 23 and 28

S30. limit 29 to (english language and humans)

________________________________

PubMed Central (1969 – April 2022) ("adult*" OR "young adult*" OR "athlete*") AND ("eccentric" OR "handheld dynamomet*" OR "dynamomet*" OR "nordic*" OR "isokinetic") AND ("reliability")

SportDiscus (EBSCOhost 1949 – July 2022)

S1. young adult

S2. adult

S3. athlet*

S4. S1 OR S2 OR S3

S5. nordic*

S6. nordic N3 exercise

S7. nordic N3 device

S8. strength N2 device

S9. eccentric N2 device

S10. eccentric N2 strength

S11. "measurement device"

S12. Nordbord

S13. "hand-held dynamomet*

S14. "digital dynamomet*

S15. Lafayette

S16. Myometer

S17. ActivForce

S18. MicroFET2

S19. Biodex

S20. Primus

S21. Cybex

S22. KinCom

S23. "Isokinetic device"

S24. S5 OR S6 OR S7 OR S8 OR S9 OR S10 OR S11 OR S12 OR S13 OR S14 OR S15 OR S16 OR S17

OR S18 OR S19 OR S20 OR S21 OR S22 OR S23

S25. reliability

S26. agreement

S27. test-retest

S28. repeated measures

S29. S25 OR S26 OR S27 OR S28

S30. S4 AND S24 AND S29

S31. S4 AND S24 AND S29 Limiters - Language: English

Google Scholar

____________________________

Grey Literature search strategies

Networked Digital Library of Theses and Dissertations (NDLTD) 1996 – July 2022

S1. Hamstring AND Eccentric – limited by English language only

Open Access Theses and Dissertations (oatd.org)

S1. hamstring OR eccentric AND reliability, limited to English only

Preprint server for Health Sciences ([medRxiv.org](http://www.medRxiv.org)) 2019 – July 2022

S1. Hamstring – Full text, Abstract, Title
