# Supplementary material for: The reliability of and agreement between devices used to measure eccentric hamstring strength: a systematic review protocol
Source: Syst Rev. 2022 Sep 23;11:204. doi: 10.1186/s13643-022-02070-8 (PMC9502956; doi:10.1186/s13643-022-02070-8)
Supplement: Supplementary file 2 — Additional file 2. PRISMA flowpart. [file 13643_2022_2070_MOESM2_ESM.docx]

Records identified during search of alternate sources - grey literature, pre-prints, snowballing methods

n=

Records identified during database search of CINAHL, EMBASE, Medlin, PubMed and SportDiscus, Google scholar, MedRxiv

n=

Records before duplicates are removed n=

Records after duplicates removed n=

Records screened based on title and abstract n=

Full articles removed with reasons n=

Articles excluded for not meeting incl/exclusion criteria n=

Identification

Records not relevant to aims and objectives of research question n=

Articles removed due to poor methodological quality n=

Article abstracts screened for eligibility n=

Screening

Articles appraised by COSMIN checklist and included in final review n=

Full-text articles receiving quality appraisal n=

Full-text articles screened for eligibility n=

Eligibility

**PRISMA Flowchart**

Included
