# Supplementary material for: The reliability of and agreement between devices used to measure eccentric hamstring strength: a systematic review protocol
Source: Syst Rev. 2022 Sep 23;11:204. doi: 10.1186/s13643-022-02070-8 (PMC9502956; doi:10.1186/s13643-022-02070-8)
Supplement: Supplementary file 3 — Additional file 3. Guide for judgement of modified GRADE synthesis. [file 13643_2022_2070_MOESM3_ESM.docx]

| GRADE DOMAIN | Explanation |
| --- | --- |
| Risk of bias | The evidence for each stated measurement device will be assigned categories of either (1) Few limitations, where most COSMIN domains are classified as low risk, (2) many limitations, where most COSMIN domains are unclear or moderate risk, or (3) very many limitations, where most COSMIN domains are high risk. |
| Imprecision | This will be evaluated by interpretation of the confidence interval width, establishing study power via sample size calculations and the number of studies that investigated each measurement device. Evidence will be categorised as precise if the confidence interval is narrow (< 0.35) or imprecise if it is wide (>0.35) [1]. |
| Inconsistency | Inconsistency relates to variability in results when two or more studies investigated comparable testing equipment, methods, and reliability statistics. Where applicable (i.e., if a meta-analysis is performed) this may be assessed by the I2. However, in the absence of a meta-analysis, inconsistency will be categorised as yes if ICCs are reported across studies according to different ICC thresholds set by Coppieters et al [2] (Poor <0.40, Moderate 0.40≤ICC<0.70, Good 0.70≤ICC<0.90, Excellent ICC≥0.90) or have different SEM measures. Evidence will be categorised as no if ICCs are reported across studies according to the same ICC thresholds as stated above and have consistent SEM values. Where only one study investigated a test and reliability this was not applicable |
| Indirectness | Indirectness is where the participant population considered by two or more comparable studies (that is, studies that investigated comparable testing equipment, methods, and reliability statistics) do not fully represent the population defined in the review question.  Evidence will be classed as direct if the study populations for two or more comparable studies are representative and generalisable to recreationally active, athletic, uninjured, or healthy adults.  Evidence will be classed as indirect if participants of two or more comparable studies are only representative of a subgroup of the population of interest (for example, male or female professional football players) |
| Publication bias | Will be considered present with all measurement devices unless there is evidence of multiple unpublished studies, studies are uniformly small, all of which may indicate ‘negative’ results |
| Overall Quality | Using the above an overall quality grade, ranging from very low quality to high quality was assigned to each measurement device and testing combination according to the methods outlined by Cochrane Handbook for Systematic Reviews of Interventions [1]. |

Supplementary File 4 – Guide for judgement of modified GRADE synthesis

1. Higgins JPT, Thomas J, Chandler J, Cumpston M, Li T, Page MJ, et al (editors). Cochrane Handbook for Systematic Reviews of Interventions version 6.3 (updated February 2022). Cochrane, 2022. <http://www.training.cochrane.org/handbook>.
2. Coppieters M, Stappaerts K, Janssens K, Jull G. Reliability of detecting ‘onset of pain’ and ‘submaximal pain’ during neural provocation testing of the upper quadrant. Physiother Res Int 2002;7(3):146-156.
